# Infectious disease, human capital, and the BRICS economy in the time of COVID-19

**DOI:** 10.1016/j.mex.2020.101202

**Published:** 2020-12-25

**Authors:** Devi Prasad Dash, Narayan Sethi, Aruna Kumar Dash

**Affiliations:** a Assistant Professor of Economics, School of Management and Entrepreneurship, Indian Institute of Technology, Jodhpur, Rajasthan, India; b Associate Professor of Economics, Department of Humanities and Social Sciences, National Institute of Technology, Rourkela, Odisha, India; c Associate Professor Economics, IBS Hyderabad, The ICFAI Foundation for Higher Education, Hyderabad, Telangana, India

**Keywords:** Population, Poverty, Infectious Disease, COVID-19, BRICS

## Abstract

We develop empirical models using difference-in-difference method to find out how COVID-19 testing and infection rates impact the BRICS economy. Our results show that strict government measures, areas of poor people and people with heart diseases have resulted in high COVID-19 testing due to the increasing infections, However, economic development and population density are not found to be rather insignificant towards the COVID-19 testing rates. Hence, both from policy and pandemic perspectives, it is inferred that these developing economies need to divert more resources and infuse more investment in the healthcare sector in the coming days.

• Governments must give due stress to the health sector along with development irrespective of nature of the economy.
• Our results show that strict government measures, areas of poor people and people with heart diseases have resulted in high COVID-19 testing due to the increasing infections.
• Both from policy and pandemic perspectives, it is inferred that these BRICS economies need to divert more resources and infuse more investment in the healthcare sector.

## Highlights

• COVID-19 has led to an economic meltdown in emerging economies such as Brazil, Russia, India, China and South Africa.
• Governments must give due stress to the health sector along with development irrespective of nature of the economy.
• Our results show that strict government measures, areas of poor people and people with heart diseases have resulted in high COVID-19 testing due to the increasing infections.
• Both from policy and pandemic perspectives, it is inferred that these BRICS economies need to divert more resources and infuse more investment in the healthcare sector.

## Graphical abstract

**Figure:**
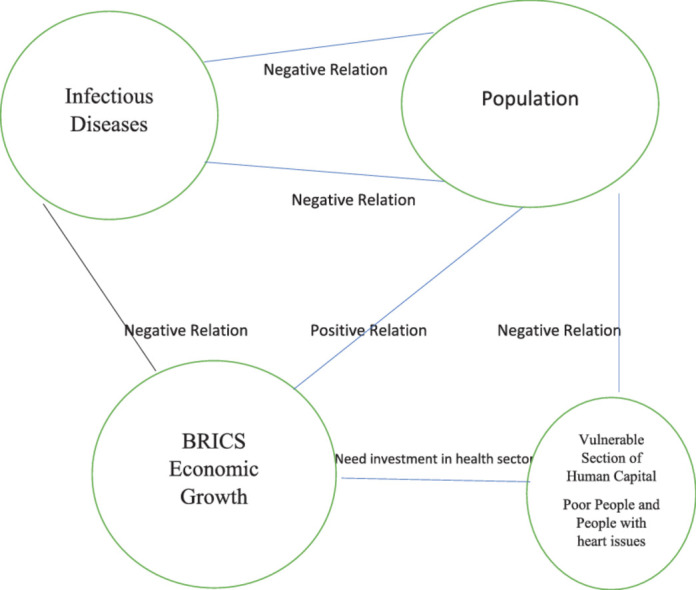

**Specifications Table**

**Table:** 

Subject Area:	Economics and Finance
More specific subject area:	Infectious Disease and Human Capital:
Method name:	difference-in-difference (DID)
Name and reference of original method:	*Bertrand, Marianne, Esther Duflo, and Sendhil Mullainathan, “How Much Should We Trust Differences-in-Differences Estimates?” Quarterly Journal of Economics, February 2004*, https://doi.org/10.1162/003355304772839588
Resource availability:	*Not Applicable*

## Method details

 

## Introduction

In this study, we investigate infectious disease, human capital, and the BRICS economy during COVID-19 scenario. COVID-19 has led to an economic meltdown in emerging economies such as Brazil, Russia, India, China and South Africa which were the engine of global growth for the past two decades. These emerging economies have already become financially vulnerable due to falling of their primary exports due to the drastic decline in global demand [28]. The economic and social impact of COVID-19 is severe for the BRICS countries. In this situation, these countries need to concentrate on steering their domestic economies out of this pandemic crisis. The pandemic has affected the manufacturing and the services sector, particularly the education, health, hotels, real estate, hospitality, tours and travel, media, IT, retails, banking sector [11]. As the pandemic spreads and the number cases increases day by day, its negative effects on the world economy are eventually becoming much more evident [30]. The COVID-19 outbreak has affected nations enormously, mainly the nationwide lockdowns that have brought the social and economic life to a virtual standstill [11]. Studies by [2], [3], [8], [10], [19], [22], [25] show that how the COVID-19 pandemic increases significantly financial market risks and uncertainty, with significant negative effects on stock returns. However, [16] found that demographic factors and government policies are significant determinants of COVID-19. Furthermore, the studies also find that ongoing pandemics have impacted world energy market substantially during this time period in terms of impacting global oil price, oil and natural gas demand. [6,^12^,^13^,^14^,^17^,23]. Present studies have also found that pandemics have impacted world economy across sectors to a greater extent negatively impacting the global outcome [21,^24^,^26^,^29^,^32^,^36^,^39^,40]. Among various sectors, pandemics have especially impacted global trade and financial sector negatively [15,^18^,^31^,^34^,38].

The COVID-19 pandemic has resulted to a tremendous loss of human life around the world and poses an enormous threat to public health and millions of the people entered into the poverty trap [4]. Adhikari et al. [1] concludes that healthcare expenditures tremendously increase along with an increase in the intensity of spreading COVID-19. Thienemann et al. [33] concludes that high population density areas increase poverty and ill patients having HIV, TB and other parasitic diseases largely affect the COVID-19. Khan et al. [20] concludes that Asian countries could hard to find the way to escape COVID 19, due to lack of access to basic amenities and vaccination make it more vulnerable situation in low-income countries while the developed countries could have a better healthcare strategy to cope the COVID-19. Walker et al. [35] quoted that COVID-19 pandemic is likely to weigh higher on a developing country because of the poor health infrastructure and surplus demand. Rollins [27] quoted that low-income earners and the people remain in poverty face difficult time to survive with the disease. Becchetti et al. [9] found a strong correlation between pollution and infection/mortality. Hence, it is important to evaluate the resilience of Brazil, Russia, India, China, and South Africa economy in presence of COVID-19.

## Methodology and data

### Methodology

The association between COVID infection rates, testing, population density and economy can be studied using a difference- in- difference (DID) method [7]. The logic behind applying DID method is to see, how the relation between outcome variable and variables of interests react through the control adopted before and after study. In this quasi-experimental design, DID is specifically used to estimate the effects of some special interventions. Since the work of [5], the DID method is used to see the relation between outcome variable and other explanatory variables in a quais-experimental design set up. In this set up especially in case of time intervention for two groups in two different time periods, we expose one of the groups to the treatment in the 2nd period, not in the 1st period. The 2nd group is not exposed to either of the period. In such instances, the same unit within the group are seen for each time period. This sort of analyses removes the biases for 2nd period comparisons between treatment and control groups and also removes the biases for comparisons in the treatment groups [37].

In our empirical strategy, intervention w.r.t time is taken especially from March 1, 2020 onwards. Intervention w.r.t space considers all 4 economies of India, China, Russia and Brazil except South Africa. This empirical setting of the study has considered the next 3 sets of equations to see the implementation of DID as an interaction between, time and treatment and time, treatment separately.

(1) $$\begin{array}{ccc} New\;test\;COVID_{I,T} & = & \alpha_{1}COVID\;Infection_{i,t}+\alpha_{2}Stringency_{i,t}+\alpha_{3}Population_{i,t}+\alpha_{4}Poverty_{i,t}\\ & & +\alpha_{5}Heart\;disease_{i,t}+\alpha_{6}\;Time+\varepsilon_{i,t}\; \end{array}$$

(2) $$\begin{array}{ccc} New\;test\;COVID_{I,T} & = & \alpha_{1}COVID\;Infection_{i,t}+\alpha_{2}Stringency_{i,t}+\alpha_{3}Population_{i,t}+\alpha_{4}Poverty_{i,t}\\ & & +\alpha_{5}Heart\;disease_{i,t}+\alpha_{6}\;Treated+\varepsilon_{i,t} \end{array}$$

(3) $$\begin{array}{ccc} New\;test\;COVID_{I,T} & = & \alpha_{1}COVID\;Infection_{i,t}+\alpha_{2}Stringency_{i,t}+\alpha_{3}Population_{i,t}+\alpha_{4}Poverty_{i,t}\\ & & +\alpha_{5}Heart\;disease_{i,t}+\alpha_{6}\;DID+\varepsilon_{i,t} \end{array}$$

In Eqs. (1), (2) and (3), we have separately treated time, treated and DID as an addition to the existing explanatory and control variables. Time in Eq. (1) considers the date prior to March 1, 2020 as zero and after March 1, 2020 as one. The rationale behind implementing Time is that most COVID-19 infections after March 1, 2020 has been increased exponentially across these 5 economies. Treated in Eq. (2) depicts the cases of India, Brazil, Russia and China as one and South Africa as zero. Keeping Brazil, India, China and Russia as one indicates that these economies have been highly impacted COVID-19 more among the developing economies. In Eq. (6), DID is used as the interaction term of both time and treated, considered earlier in both Eq. (3). Similarly, for sensitivity analysis, DID regressions are also being framed by considering GDP in place of population to see, how changes in economic performance of these economies impact the COVID19 testing rates.

(4) $$\begin{array}{ccc} New\;test\;COVID_{I,T} & = & \alpha_{1}COVID\;Infection_{i,t}+\alpha_{2}Stringency_{i,t}+\alpha_{3}GDP_{i,t}+\alpha_{4}Poverty_{i,t}\\ & & +\alpha_{5}Heart\;disease_{i,t}+\alpha_{6}\;Time+\varepsilon_{i,t} \end{array}$$

(5) $$\begin{array}{ccc} New\;test\;COVID_{I,T} & = & \alpha_{1}COVID\;Infection_{i,t}+\alpha_{2}Stringency_{i,t}+\alpha_{3}GDP_{i,t}+\alpha_{4}Poverty_{i,t}\\ & & +\alpha_{5}Heart\;disease_{i,t}+\alpha_{6}\;Treated+\varepsilon_{i,t} \end{array}$$

(6) $$\begin{array}{ccc} New\;test\;COVID_{I,T} & = & \alpha_{1}COVID\;Infection_{i,t}+\alpha_{2}Stringency_{i,t}+\alpha_{3}Population_{i,t}+\alpha_{4}Poverty_{i,t}\\ & & +\alpha_{5}Heart\;disease_{i,t}+\alpha_{6}\;DID+\varepsilon_{i,t} \end{array}$$

### Data

In this study, we empirically examine the relation between COVID infection, government measures and economic growth for BRICS economies over the time period Jan 1, 2020 to Oct 1, 2020. In BRICS economies, we have taken the usual 5 economies- India, China, Russia, Brazil and South Africa. We have considered numbers of COVID tests as the outcome variable. New COVID-19 infection, population, GDP and stringency are considered as the major explanatory variables. Poverty and Number of people with heart disease are considered as the control variables of the study. All the data pertaining to our study are taken from Ourworldindata on a daily frequency basis. New COVID-19 testing infection is defined as the numbers of testing for people to detect COVID on a daily basis. New COVID-19 infection is defined as the numbers of people tested positive for COVID on a daily basis. Population is defined as the total number of persons both in urban and rural areas on a daily basis. Population is also considered as the unique proxy for the market size. GDP is defined as the final goods and services produced in the economy. Stringency in this case is defined as the stringent measures taken by the respective government in terms of curbing the movement of the people, economic activities, and shutdown measures as well. Stringency index is here measured in a scale ranging from 0 to 100. Furthermore, the control variables in this empirical model are poverty and people with heart diseases. The rationale behind taking the variables implies that people mostly in poor conditions and with pre-existing diseases like heart diseases are highly vulnerable to COVID.

### Empirical findings

Table-1 shows the DID regression results in presence of the major explanatory variables population. In case of time specific intervention, we notice that stringency has especially worked well in terms of increasing COVID testing rates over the days. Our empirical results also suggest that every 1% increase in stringency measures has led to nearly 0.29% increase in the COVID-19-testing rates. More so, coefficients of poverty and heart disease and time are steadfastly indicating positive and significant association with the testing rates over the days. In cases of considering country specific interventions, we notice that except population, other coefficients exhibit positive and significant relation with the COVID testing rates. However, we find that coefficients of treated and population are found to be negatively and significantly associated with the testing rates over the days.

Next, we have considered GDP as one of the explanatory variables. InTable 2, our empirical results show that every 1% increase in COVID-19 infection rates has led to the increase in testing rates by 0.632% to 0.704% over the days. In cases of stringency measures, we notice that coefficients of stringency show positive and significant result in cases of country specific interventions. However, w.r.t development, stringency has not worked well in case of time specific intervention. It is due to the under-reporting of the COVID-19 testing rates in the developing economies.

**Table 1:** DID estimation.

New test COVID	I	II	III
New COVID infection	0.584* (0.027)	0.709* (0.031)	0.619* (0.029)
Stringency	0.290* (0.108)	0.456* (0.085)	0.789* (0.080)
Population	−1.187* (0.059)	−0.653* (0.101)	−1.169* (0.063)
Poverty	2.516* (0.067)	2.066* (0.093)	2.468* (0.073)
Heart Disease	1.461* (0.366)	1.624* (0.426)	1.577* (0.269)
Time	0.784* (0.134)		
Treated		−1.177* (0.160)	
DID			−0.269* (0.099)
Constant	−2.607* (0.755)	−2.243* (0.921)	−2.093* (0.814)
*R*^2^	0.796	0.799	0.792

***Notes:*** Author's own compilation. ***p < 0.10; **p < 0.05; *p < 0.01. All variables are converted into natural logarithm.

**Table 2:** DID Regression with GDP.

New test COVID	I	II	III
**New COVID infection**	0.632* (0.026)	0.709* (0.031)	0.654* (0.028)
**Stringency**	0.150 (0.104)	0.456* (0.085)	0.614* (0.078)
**GDP**	0.797* (0.311)	4.813* (0.754)	1.068* (0.383)
**Poverty**	0.597* (0.122)	3.708* (0.320)	4.697* (0.156)
**Heart Disease**	1.233* (0.260)	1.412* (0.264)	1.309* (0.264)
**Time**	0.813* (0.132)		
**Treated**		-0.760* (0.216)	
**DID**			-0.054 (0.051)
**Constant**	-6.618* (1.624)	4.649* (3.509)	-5.095* (1.809)
***R^2^***	0.803	0.799	0.797

***Notes:*** Author's own compilation. ***p < 0.10; **p < 0.05; *p < 0.01. All variables are converted into natural logarithm.

## Conclusion

This study documents the rapid emergence of the COVID-19 infection rates at the onset of rising economic development and several economic challenges. In our empirical experiments, we also find that areas with more economic development and high population density are often exhibiting lesser COVID testing incidents due to the lack of co-operation and under-reporting of COVID testing. Specifically, our empirical investigation shows that although testing rates have been increased positively with the infection rates, still people with lesser economic likelihoods are highly exposed to the coronavirus in these countries.

This study has led to some policy suggestions. First, the developing economies must invest more of its resources towards the healthcare sector. Second, more of testing to common people along with opening hospital avenues can help curb and detect the exposure of common masses.

## Declaration of Competing Interest

The authors declare that they have no known competing financial interests or personal relationships that could have appeared to influence the work reported in this paper.
